# Behavior and interaction imaging at 9 months of age predict autism/intellectual disability in high-risk infants with West syndrome

**DOI:** 10.1038/s41398-020-0743-8

**Published:** 2020-02-03

**Authors:** Lisa Ouss, Giuseppe Palestra, Catherine Saint-Georges, Marluce Leitgel Gille, Mohamed Afshar, Hugues Pellerin, Kevin Bailly, Mohamed Chetouani, Laurence Robel, Bernard Golse, Rima Nabbout, Isabelle Desguerre, Mariana Guergova-Kuras, David Cohen

**Affiliations:** 1 Service de Psychiatrie de l’Enfant, AP-HP, Hôpital Necker, 149 rue de Sèvres, 75015 Paris, France; 2 Institut des Systèmes Intelligents et de Robotique, CNRS, UMR 7222, Sorbonne Université, 4 Place Jussieu, 75252 Paris Cedex, France; 3 Département de Psychiatrie de l’Enfant et de l’Adolescent, AP-HP, Hôpital Pitié-Salpêtrière, 47-83, Boulevard de l’Hôpital, 75651 Paris, Cedex 13 France; 4 Ariana Pharmaceuticals, Research Department, Paris, France; 5 Service de Neuropédiatrie, AP-HP, Hôpital Necker, 136, Rue de Vaugirard, 75015 Paris, France

**Keywords:** Predictive markers, Autism spectrum disorders

## Abstract

Automated behavior analysis are promising tools to overcome current assessment limitations in psychiatry. At 9 months of age, we recorded 32 infants with West syndrome (WS) and 19 typically developing (TD) controls during a standardized mother–infant interaction. We computed infant hand movements (HM), speech turn taking of both partners (vocalization, pause, silences, overlap) and motherese. Then, we assessed whether multimodal social signals and interactional synchrony at 9 months could predict outcomes (autism spectrum disorder (ASD) and intellectual disability (ID)) of infants with WS at 4 years. At follow-up, 10 infants developed ASD/ID (WS+). The best machine learning reached 76.47% accuracy classifying WS vs. TD and 81.25% accuracy classifying WS+ vs. WS−. The 10 best features to distinguish WS+ and WS− included a combination of infant vocalizations and HM features combined with synchrony vocalization features. These data indicate that behavioral and interaction imaging was able to predict ASD/ID in high-risk children with WS.

## Introduction

Behavior and interaction imaging is a promising domain of affective computing to explore psychiatric conditions^1–3^. Regarding child psychiatry, many researchers have attempted to identify reliable indicators of neurodevelopmental disorders (NDD) in high-risk populations (e.g., siblings of children with autism) during the first year of life to recommend early interventions^4,5^. However, social signals and any alterations of them are very difficult to identify at such a young age^6^. In addition, exploring the quality and dynamics of early interactions is a complex endeavor. It usually requires (i) the perception and integration of multimodal social signals and (ii) an understanding of how two interactive partners synchronize and proceed in turn taking^7,8^.

Affective computing offers the possibility to simultaneously analyze the interaction of several partners while considering the multimodal nature and dynamics of social signals and behaviors^9^. To date, few seminal studies have attempted to apply social signal processing to mother–infant interactions with or without a specific condition, and these studies have focused on speech turns (e.g., Jaffe et al.^10^), motherese^11^, head movements^12^, hand movements^13^, movement kinematics^2^, and facial expressions^3^.

Here, we focused on West syndrome (WS), a rare epileptic encephalopathy with early onset (before age 1 year) and a high risk of NDD outcomes, including one-third of WS children showing later autism spectrum disorder (ASD) and/or intellectual disability (ID). We recruited 32 infants with WS and 19 typically developing (TD) controls to participate in a standardized early mother–infant interaction protocol and followed infants with WS to assess outcomes at 4 years of age. We aim to explore whether multimodal social signals and interpersonal synchrony of infant–mother interactions at 9 months could predict outcomes.

## Materials and methods

### Design, participants, and clinical measures

We performed a prospective follow-up study of infants with WS^14^. The Institutional Review Board (*Comité de Protection des Personnes* from the *Groupe-Hospitalier Necker Enfants Malades*) approved the study, and both parents gave written informed consent after they received verbal and written information on the study. They were asked to participate to a follow-up study to assess outcome of WS taking into account development, early interaction, genetics and response to pharmacological treatment^14^. The study was conducted from November 2004 to March 2010 in the Neuro-pediatrics Department Center for Rare Epilepsia of *Necker Enfants-Malades* Hospital, Paris. Of the 41 patients screened during the study period, we enrolled all but two cases (*N* = 39) with WS. Seven patients dropped out before the age of 3 leading to a sample of 32 patients with detailed follow-up data. Typical developing infants (*N* = 19) were recruited from Maternal and Infant Prevention institutions, in pediatric consultations, or by proxy.

To assess neurodevelopmental outcomes, we focused on ID and ASD. ID was assessed through the *Brunet-Lézine Developmental Examination*, performed for all children at the age of 3 years. The Brunet-Lézine Developmental Examination estimates a developmental quotient (DQ) based upon normative data available for 3-year-old French toddlers^15^. The diagnosis of autism was based upon several measurements and an expert assessment that was blind to other variables: (i) At 3 years of age, all parents completed the *Autism Diagnostic Interview-Revised* (ADI-R) to assess autism signs by dimensions and developmental delay^16^. (ii) At 2 and 3 years of age, all patients were assessed with the *Children’s Autism Rating Scale* (CARS)^17^. (iii) An expert clinician (LR) who was blind to child history assessed autism and ID from 20-min videotapes of child/mother play at 2 years of age. Finally, diagnoses of ASD and/or ID at age 4 were based upon a consensus approach using direct assessment of the child by a clinician with expertise in autism (LO) as well as by clinical information from the CARS, ADI-R, and DQ.

### Video recordings

**Fig. 1:**
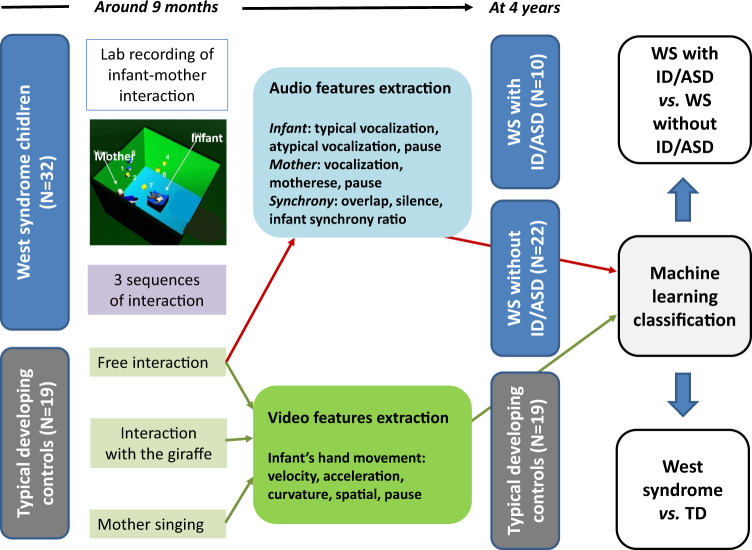
Pipeline of our machine learning approach to classify WS vs. TD.

Infant–mother interactions were assessed between 9 and 12 months of age during a play session (Fig. 1). Two synchronized cameras (face and profile; Fig. S1A) recorded the movements in two dimensions while the infant was sitting in a baby chair. Audio interactions were also recorded. The standardized situation encompassed three sequences of 3 min: (sequence 1) free play after instructing the mother to interact “as usual” without any toy; (sequence 2) free play using the help of a toy (Sophie the giraffe); (sequence 3) mother singing to her baby. Due to the position of the baby chair on the floor and the mother’s seated position, the mother was positioned slightly higher in all of the recordings. The mother’s indicated position was on the left of the child as shown on the picture, but exceptions were sometimes observed during the recordings. For infant hand movement (HM) features, 1 min was extracted from each 3-min video and all recordings, according to two criteria: the child’s hands should be visible for at least part of the sequence (e.g., the mother is not leaning on the child), and the minute represented the greatest amount of interaction between the mother and the child. For audio and speech turn-taking computing, we only used the 3-min audio recording of sequence 1.

### Vision computing (Fig. S1B, vision computing panel)

To process infant hand movements (HM), we used the methods developed in Ouss et al.^13^. Here, we summarize the successive steps to calculate HM features. In step 1 (hand trajectory extraction and data processing), the two-dimensional coordinates of the hand were extracted from each of the video recordings by tracking a wristband on the right hand (yellow in Fig. S1A, video-audio recording panel). The tracking framework comprised three steps: prediction, observation, and estimation as proposed in ref. ^18^. As the hand motion was highly nonlinear, we developed an approach using a bootstrap-based particle filter with a first-order model to address abrupt changes in direction and speed^19,20^. To address hand occlusion, we implemented an approach combining tracking with detection by adding a boolean variable to the state vector associated with each particle^18^.

Each extracted trajectory consisted of 1500 pairs of *x* and *y* coordinates (25 frames per second, generating 1500 pairs of coordinates in the 60 s; see Fig. S1 left panel, vision computing). The frames where the hand was not visible were clearly indicated in each trajectory as missing coordinates for these time points. To account for differences in the camera zoom parameters, the trajectories obtained were normalized using a fixed reference system present in the settings of each video recording. The normalization was performed on all trajectories, and 95% of the normalization factors ranged between 0.8 and 1.22 with a few outlier trajectories that required greater correction. Forty-one percent of the trajectories required <5% correction. Although the recordings between the two cameras were synchronized and in principle allowed 3D reconstruction of the trajectory, the accumulation of missing data prevented such reconstruction. However, 2D motion capture with appropriately defined movement descriptors can be powerful for detecting clinically relevant changes^21^, thereby justifying the independent analysis of the 2D-trajectory videos (see Fig. S1B, vision computing, 2d panel on the left).

In step 2, the descriptors of the HM were calculated from the planar trajectories (Fig. S1B, table shown in the vision computing panel). Descriptors covered those already reported in the literature as important in characterizing infants’ HM^21^. (1) To describe the space explored by the hand, we calculated the maximum distance observed on the two axes (xRange, yRange) and the standard deviation of the X and Y coordinates observed during the 60 s (xSd, ySd). We also calculated the maximum distance between any two points of the trajectory using the FarthestPair java library (http://algs4.cs.princeton.edu/code/) (Fig. S1B, vision computing panel, red line in the third panel from the left). (2) To evaluate HM dynamics, we calculated the velocity and acceleration. (3) Also related to HM dynamics, we calculated HM pauses defined as part of the trajectory in which the velocity was lower than a specific threshold for a minimum duration of 4 s. (4) Finally, the curvature of the trajectories was calculated using a standard definition of the curvature (*κ*) of plane curves in Cartesian coordinates as *γ*(*t*) = (*x*(*t*), *y*(*t*)). The curvature calculated at each point of the trajectory is presented in the right panel of Fig. S1B (video computing), where the first 1.2 s of the trajectory are plotted and the associated calculated curvatures at each point (and respective time, indicated on the axis) are presented as columns.

### Audio computing (Fig. S1C, audio computing)

We extracted two types of audio social signals from the audio channel of the mother–infant interaction: speech turn taking (STT) and motherese. For STT extraction, we followed the methods developed by Weisman et al.^22^ and Bourvis et al.^23^ (Fig. S1, audio computing). First, we used ELAN to segment the infants’ and mothers’ speech turns and annotate the dialog acts. Mothers’ audio interactions were categorized as mother vocalization (meaningful vocalizations, laugh, singing, animal sounds) or other noise (clap hands, snap fingers or snap the tongue, mouth noise, etc.). Similarly, infants’ audio production was defined as infant vocalization (babbling vocalizations, laugh, and cry) or atypical vocalization (other noise such as “rale”). The infant’s and mother’s utterances were labeled by two annotators (blind to group status). Cohen’s kappa between the two annotators was calculated for each dyad, each task and each item of the grid. For all items, the kappa values were between 0.82 and 1.

From the annotation, we extracted all the speech turns of the infant and the mother. A speech turn is a continuous stream of speech with <150 ms of silence. We obtained a list of triples: speaker label (infant or mother), start time, and duration of speech turn. From these triples, we also deduced the start time and duration of the time segments when the mother or the infant were not speaking (pauses). Therefore, we extracted M*other Vocalizations*; *Mother Other Noise*; *Infant Vocalizations*; *Infant Atypical Vocalizations*; *Mother Pauses*; *Infant Pauses*. We also extracted three dyadic features: (1) *Silence* defined as sequences of time during which neither participant was speaking for more than 150 ms; (2) *Overlap Ratio* defined as the duration of vocalization overlaps between mothers and infants divided by the duration of the total interaction. This ratio measures the proportion of interactional time in which both participants were simultaneously vocalizing; (3) *Infant Synchrony Ratio* defined as the number of infants’ responses to their mother’s vocalization within a time limit of 3 s divided by the number of mother vocalizations during the time paradigm. The 3-s window was based on the available literature on synchrony^7,24^.

From the mother vocalizations, we also computed affective speech analysis, as previous work has shown that motherese may shape parent-infant interactions^25^. The segments of mother vocalizations were analyzed using a computerized classifier for categorization as “motherese” or “non-motherese/other speech” initially developed to analyze home movies^11^. The system exploits the fusion of two classifiers, namely, segmental and suprasegmental^26^. Consequently, the utterances are characterized by both segmental (Mel frequency cepstrum coefficients) and suprasegmental/prosodics (e.g., statistics with regard to fundamental frequency, energy, and duration) features. The detector used the GMM (Gaussian mixture model) classifier for both segmental and suprasegmental features (*M*, number of Gaussians for the GMM Classifier: *M* = 12 and 15, respectively, and *λ* = weighting coefficient used in the equation fusion: *λ* = 0.4). For the purpose of the current study, we explored the performance of our motherese classifier in French mothers. We analyzed 200 sequences from French mothers (100 motherese vs. 100 other speech) that were blindly validated by two psycholinguists. We calculated the Intraclass correlation (ICC) between the two raters (the expert and the algorithm) and found a good and very significant ICC (ICC = 0.79 (95% CI: 0.59–0.90), *p* < 0.001). This level of prediction made it suitable for further analysis of the entire data set.

Based on this automatic detection of motherese, we created two subclasses for mother vocalizations: motherese vs. non-motherese. Two variables were derived: *Motherese Ratio* (duration of motherese vocalization/duration of interaction) and *Non-motherese Ratio* (duration of non-motherese vocalization/duration of interaction). We also derived two synchrony ratios: *Synchrony Motherese Ratio* and *Synchrony Non-motherese Ratio*, which reflect the ratio of time during which the infant vocalizes in response to his/her mother motherese and other speech (non-motherese).

### Prediction of the outcome using machine learning

The pipeline of our approach is shown in Fig. 1. First, a data quality analysis was performed to ensure the validity of the data. As expected, all data were available for audio analysis. However, a substantial proportion of the data were discarded due to video recording or vision computing issues. We finally kept 18 video recordings for the WS and 17 videos for the TD groups. Second, given the number of features (21 infant HM for each camera and each sequence; 16 STT) compared with the data set (32 WS and 19 TD), we reduced our data using principal component analysis (PCA). Third, we tested several algorithms to classify WS vs. TD based on the whole data set available for both vision and audio computing features (leave one out) (Table S1). The best algorithm was decision stump^27^. All results presented here are based on the classification with a decision stump algorithm. We also analyzed WS with ID/ASD (WS+) vs. WS without ID/ASD (WS−). For each classification, we also extracted a confusion matrix and explored which individual features contributed the most to a given classification using Pearson correlations.

## Results

Table S2 summarizes the demographic and clinical characteristics of children with WS. At follow-up, 10 infants out of 32 children with WS developed ASD/ID (WS+). Eight children had ASD and ID, whereas 2 had only ID. As expected, all variables related to ASD and ID were significantly different in WS+ compared with WS−.

**Fig. 2:**
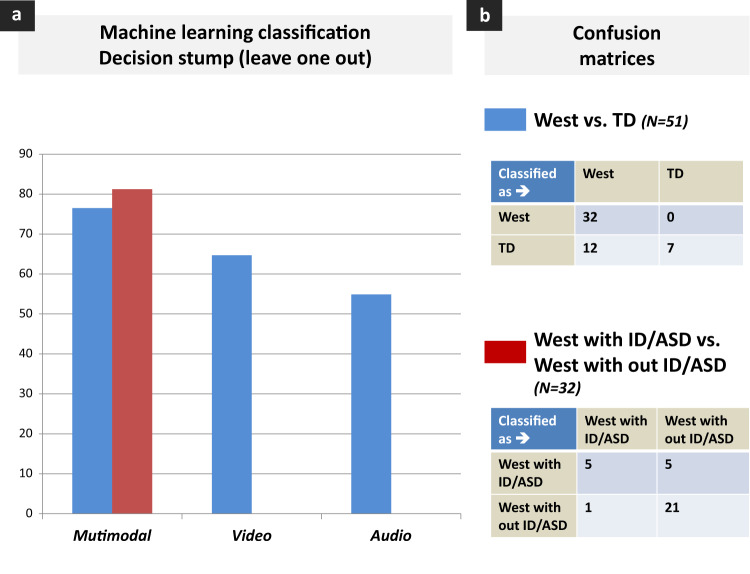
Machine learning classification of WS vs. TD and WS+ vs. WS− based on uni- and multimodal features extracted during early infant–mother interaction.

Figure 2a summarizes the best classification models using the decision stump algorithm (leave one out). As shown, multimodal classification outperformed unimodal classification to distinguish WS and TD. Therefore, we only used the multimodal approach to classify WS+ vs. WS−. The best model reached 76.47% accuracy classifying WS vs. TD and 81.25% accuracy classifying WS+ vs. WS− based on multimodal features extracted during early interactions. Interestingly, the confusion matrices (Fig. 2b) show that when classifying WS vs. TD, all errors came from TD being misclassified as WS (*N* = 12); when classifying WS+ vs. WS−, most errors came from WS+ being misclassified as WS− (*N* = 5).

Table 1 lists the best features for each multimodal classification based on the Pearson correlation values. The best features to distinguish WS and TD included four infant HM features, 1 mother audio feature. In contrast, the best features to distinguish WS+ and WS− included a combination of infant vocalization features (*N* = 2), synchrony vocalization features (*N* = 3) and infant HM features (*N* = 5), the last of which showed lower correlation scores.

**Table 1:** Best features for classification (based on significant Pearson’s correlation between feature and class).

	Feature characteristics	Pearson *r*	*p*-value
West vs. Typical developing			
Ratio of all maternal audio intervention during free interaction	Audio, mother	0.35	0.012
Total number of infant HM pauses (side view camera) during free interaction	Video, infant	0.34	0.014
Total number of infant HM pauses (side view camera) when the mother is singing	Video, infant	0.32	0.023
Vertical amplitude of the giraffe (front view camera)	Video, infant	−0.30	0.032
Movement acceleration max (side view camera) during free interaction	Video, infant	0.29	0.034
West with ASD/ID vs. West without ASD/ID			
Total number of all infant vocalization during free interaction	Audio, infant	−0.56	<0.001
Synchrony ratio (infant response to mother)	Audio, synchrony	−0.55	<0.001
Ratio of all infant vocalization during free interaction	Audio, infant	−0.55	0.001
Motherese synchrony ratio (infant response to motherese)	Audio, synchrony	−0.54	0.002
Non-motherese synchrony ratio (infant response to non-motherese)	Audio, synchrony	−0.48	0.005
HM acceleration SD (front view camera) during the giraffe interaction	Video, infant	−0.46	0.008
HM acceleration max (side view camera) during the giraffe interaction	Video, infant	−0.45	0.01
HM velocity SD (front view camera) during the giraffe interaction	Video, infant	−0.43	0.014
Curvature max (side view camera) during the giraffe interaction	Video, infant	−0.37	0.039
Relative time spent motionless (pause) (front view camera) during free interaction	Video, infant	0.36	0.04

*HM* hand movement, *ASD* autism spectrum disorder, *ID* intellectual disability, *SD* standard deviation.

## Discussion

To the best of our knowledge, this is the first study to apply multimodal social signal processing to mother–infant interactions in the context of WS. Combining speech turns and infant HM during an infant–mother interaction at 9 months significantly predicted the development of ASD or severe to moderate ID at 4 years of age in the high-risk children with WS. Confusion matrices showed that the classification errors were not random, enhancing the interest of the computational method proposed here. In addition, the best contributing features for the performed classifications differed when classifying WS vs. TD and WS+ vs. WS−. Infant HMs were the most significant features to distinguish WS versus TD, probably reflecting the motor impact due to acute WS encephalopathy. For classifying WS+ vs. WS−, the contribution of infant audio features and synchrony features became much more relevant combined with several HM features.

We believe that the importance of synchrony and reciprocity during early interactions is in line with recent studies that have investigated the risk of ASD or NDD during the first year of life from home movies (e.g., refs. ^11,24^), from prospective follow-up of high-risk infants such as siblings (e.g., refs. ^4,28^) or infants with WS (e.g., ref. ^14^), and from prospective studies assessing tools to screen risk for autism (e.g., ref. ^29^). In the field of ASD, synchrony, reciprocity, parental sensitivity, and emotional engagement are now proposed as targets of early interventions^30^, which could prevent early interactive vicious circles. Parents of at-risk infants try to compensate for the lack of interactivity of their child by modifying their stimulation and therefore sometimes reinforcing the dysfunctional interactions^24^. Early identification of these interactive targets is especially useful among babies with neurological comorbidities because delays in developmental milestones and impairments in early social interactions are not sufficient to predict ASD.

Similarly, we believe that the importance of HM in distinguishing WS vs. TD on one hand, and WS+ vs. WS− on the other hand, is also in line with the studies that investigated the importance of non-social behaviors for investigating the risk of ASD or NDD during the first year of life. For example, studying home movies, Purpura et al. found more bilateral HM and finger movements in infants who will later develop ASD^31^. Similarly, several prospective follow-up studies of high-risk siblings^32–35^ or retrospective studies on home movies^36,37^ reported specific motor atypical repertoire in infants with ASD.

In ASD, early social signals have previously been assessed with automatized and computational procedures, focusing on eye tracking at early stages^38–40^, vocal productions^41^, analysis of acoustics of first utterances or cry episodes^42^, but none was done in an interactive setting. Our study proposed a paradigm shift from the assessment of infant behavior to dyadic assessment of interactions, as previously achieved in retrospective approaches using home movies^24^. The aim is not to implement studies of social signal processing in routine clinical work but rather to decompose clinical intuitions and signs and validate the most relevant cues of these clinical features. From clinical work, back to clinics, social signal processing is a rigorous step to help clinicians better identify and assess early targets of interventions.

Given the exploratory nature of both our approach and method, our results should be interpreted with caution taking into account strengths (prospective follow-up, automatized multimodal social signal processing, and ecological standardized assessment) and limitations. These limitations include (1) the overall sample size knowing that WS is a rare disease; (2) the high rate of missing data during video recording due to the ecological conditions of the infant–mother interaction (mothers interposing between the camera and the infant); the final sample size of WS+ (*N* = 10) that limited the power of machine learning methods.

We conclude that the method proposed here combining multimodal automatized assessment of social signal processing during early interaction with infants at risk for NDD is a promising tool to decipher clinical features that remain difficult to identify and assess. In the context of WS, we showed that such a method we proposed to label ‘behavioral and interaction imaging’ was able to significantly predict the development of ASD or ID at 4 years of age in high-risk children who had WS and were assessed at 9 months of age.

## Supplementary information

Supplementary material figure S1

Supplementary material Table S1 and S2
