# Supplementary material for: Behavior and interaction imaging at 9 months of age predict autism/intellectual disability in high-risk infants with West syndrome
Source: Transl Psychiatry. 2020 Feb 3;10:54. doi: 10.1038/s41398-020-0743-8 (PMC7026100; doi:10.1038/s41398-020-0743-8)
Supplement: Supplementary file 1 — Supplementary material figure S1 [file 41398_2020_743_MOESM1_ESM.pdf]

A. Video-audio recording

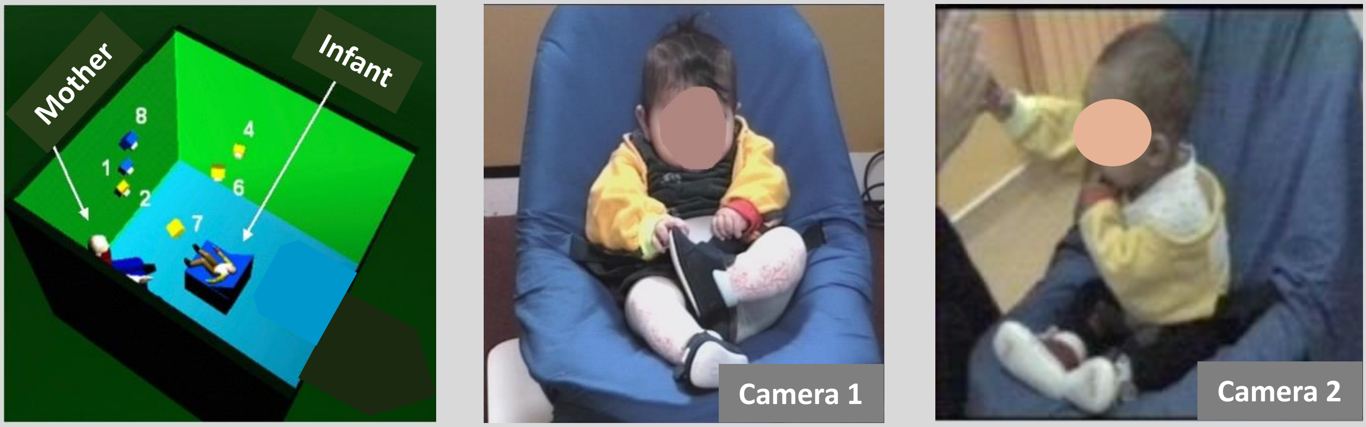

B. Vision computing

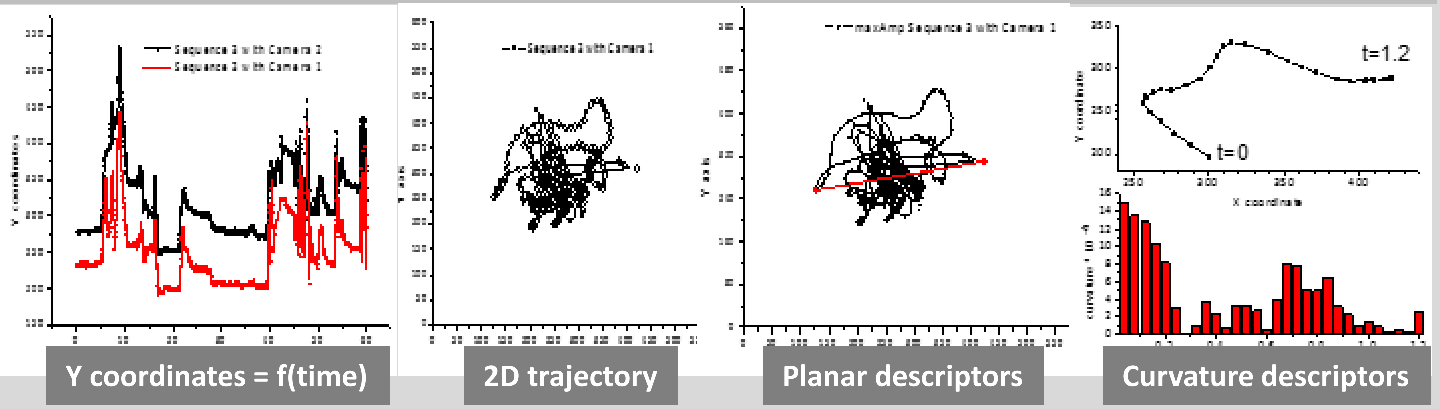

Variable	Features
Velocity	Mean, SD, Max, Min
Acceleration	Mean, SD, Max, Min
Curvature	Mean, SD, Max
Spatial	Y Range, X Range, X SD, Y SD, Amplitude Max
Pause	N, Mean, Max, Min, %

C. Audio and speech turn taking computing

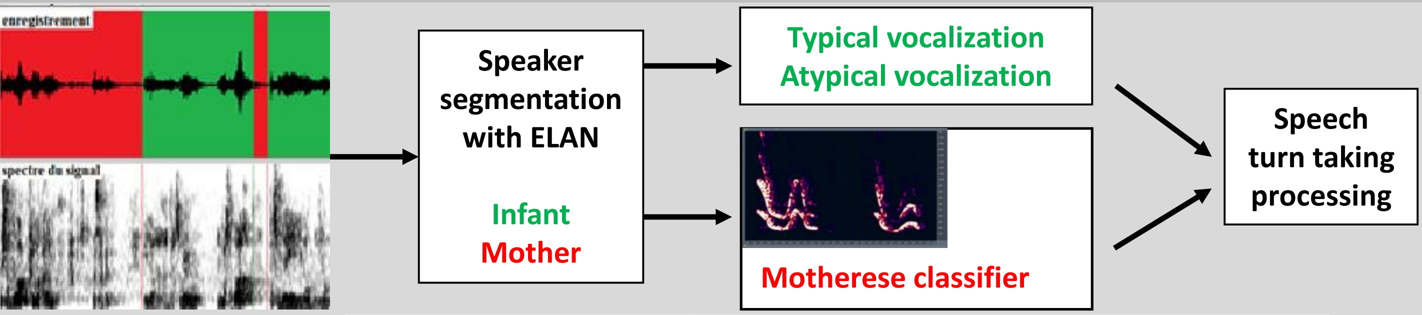

Variable	Features
Infant	Typical vocalization (Mean, SD)<br>Atypical vocalization (Mean, SD)<br>Pause (Mean, SD)
Mother	Vocalization (Mean, SD)<br>Motherese (%, ratio)<br>Pause (Mean, SD)
Dyadic	Overlap (Mean, SD)<br>Silence (Mean, SD)<br>Infant synchrony ratio (Mean, SD)
