# Supplementary material for: Behavior and interaction imaging at 9 months of age predict autism/intellectual disability in high-risk infants with West syndrome
Source: Transl Psychiatry. 2020 Feb 3;10:54. doi: 10.1038/s41398-020-0743-8 (PMC7026100; doi:10.1038/s41398-020-0743-8)
Supplement: Supplementary file 2 — Supplementary material Table S1 and S2 [file 41398_2020_743_MOESM2_ESM.pdf]

Behavior and interaction imaging at 9 months of age predict autism/intellectual disability in high-risk infants

Supplementary Tables

Table S1. Comparison of several algorithms to classify WS vs. TD after principal component analysis

	accuracy	t-test	p-value
PCA + Decision Stump (27)	76.47%	reference	reference
PCA + LibSVM (36)	62.74%	-8.828	<0.00001
PCA + J48 (37)	64.71%	-3.0399	0.0029
PCA + Random classification (ZeroR) (38)	62.74%	-8.8315	<0.00001

Table S2. Characteristics of the children with West syndrome prospectively followed until 4 years of age (N=32)

Sex: N (%)	Female: 19 (59.4); Male: 13 (40.6)	
Mother education: N (%)	Graduate: 17 (53.1); Undergraduate: 15 (46.9)	
Father education: N (%)	Graduate: 16 (50); Undergraduate: 16 (50)	
Medical condition: N (%)	Tuberous Sclerosis: 6 (18.7); Structural: 7 (21.9); Infection: 1 (3.1); Genetic/metabolic: 3 (9.4); Unknown: 15 (46.9)	
Psychiatric diagnosis at follow-up: N (%)	Autism: 8 (25); ID without autism: 2 (6.25)	
	Subjects without ASD/ID	Subjects with ASD/ID only
DQ at age 3: mean (SD) [range]	79.18 (18.1) [57-105]	29.82 (15.37) [9-55]
CARS at age 2: mean (SD) [range]	18.85 (2.67) [15.5-23.5]	35.55 (7.59) [18-42.5]
ADI-R social score: mean (SD) [range]	1.31 (1.6) [0-4]	14.5 (7.72) [0-24]
ADI-R communication score: mean (SD) [range]	1.38 (1.5) [0-4]	10.0 (4.31) [0-14]
ADI-R stereotypies score: mean (SD) [range]	0.38 (0.65) [0-2]	3.73 (2.45) [2-8]
ADI-R developmental score: mean (SD) [range]	2.62 (1.39) [0-5]	4.73 (0.6) [4-5]

ID: intellectual disability; DQ: development quotient; CARS: Children Autism Rating Scale; ADI-R: Autism Diagnostic Interview-Revised
